# Pregnancy outcomes in women with Celiac disease in Northeast Iran: a regional retrospective cohort study

**DOI:** 10.1186/s12876-024-03325-5

**Published:** 2024-07-23

**Authors:** Saeed Sahebdel, Azita Ganji, Sajedeh Alijan Nezhad Baei, Malihe Amirian, Ehsan Mousa Farkhani, Mohammad Ebrahimi, Eisa Nazar, Mohammad Amin Khojastehnezhad, Sadaf Valizadeh

**Affiliations:** 1 Student Research Committee, Faculty of Medicine, Mashhad University of Medical Sciences, Mashhad, Iran; 2 Department of Gastroenterology and Hepatology, Faculty of Medicine, Mashhad University of Medical Sciences, Mashhad, Iran; 3 Cancer Research Center, Mashhad University of Medical Sciences, Mashhad, Iran; 4 Department of Obstetrics and Gynecology, School of Medicine, Mashhad University of Medical Sciences, Mashhad, Iran; 5 Department of Epidemiology, Faculty of Health, Mashhad University of Medical Sciences, Mashhad, Iran; 6 Orthopedic Research Center, Mazandaran University of Medical Sciences, Sari, Iran; 7 Department of Internal Medicine, Faculty of Medicine, Islamic Azad University of Mashhad, Mashhad, Iran; 8 Department of Biology, Faculty of Basic Sciences, Azarbaijan Shahid Madani University, Tabriz, Iran

**Keywords:** Celiac disease, Pregnancy outcomes, Miscarriage, Preeclampsia, Low birth weight

## Abstract

**Purpose:**

To investigate the odds and associations of pregnancy outcomes with exposure to biopsy-confirmed celiac disease (CD) in Northeast Iran.

**Methods:**

In this regional retrospective cohort study, pregnancy records of all women with celiac disease who visited Celiac Disease Clinic of Imam-Reza Hospital from 2017 to 2023 (exposed group) and a sample of women without CD (unexposed group) were extracted using the Electronic Health Record of Mashhad University of Medical Sciences called “Sina”. The unexposed group was randomly selected of the database and matched to exposed group on age, location of residence, socioeconomic factors. Our exclusion criteria included age ≥ 45, presence of concomitant disorders, history of non-obstetric uterine surgery, induction of pregnancy through assisted reproductive technology, and any concurrently ongoing pregnancy at the time of study. Pregnancy outcomes evaluated in this study included normal delivery, miscarriage, preterm labor, preeclampsia, and stillbirth. Adjusted odds ratios were calculated using logistic regression adjusted for confounders.

**Results:**

Ninety pregnancy records of women with CD and 270 pregnancies of women without CD were included in this study. Low neonatal birthweight (i.e. under 2500 g) had no significant association with CD (aOR = 0.99, 95% CI = 0.92–1.06), as well as postpartum hemorrhage (aOR = 1.12, 95%CI = 0.91–1.38), fetal anomaly (aOR = 0.89, 95%CI = 0.69–1.15), miscarriage (aOR = 1.00, 95%CI = 0.91–1.10), ectopic pregnancy (aOR = 0.94, 95%CI = 0.73–1.20), preterm labor (aOR = 1.00, 95%CI = 0.92–1.10), gestational diabetes mellitus (aOR = 1.07, 95%CI = 0.98–1.16), gestational hypertension (aOR = 0.99, 95%CI = 0.89–1.11), and gestation hypothyroidism (aOR = 0.95, 95%CI = 0.82–1.11). However, we found significantly lower odds of preeclampsia in pregnancies affected by CD (aOR = 0.83, 95%CI = 0.69–0.99).

**Conclusion:**

Celiac disease was not associated with increased odds of low neonatal birthweight, postpartum hemorrhage, fetal anomaly, miscarriage, ectopic pregnancy, preterm labor, gestational diabetes mellitus, gestational hypertension and gestational hypothyroidism. Preeclampsia had significantly lower odds in pregnancies affected with CD.

## Introduction

Celiac disease (CD) is a long-lasting hypersensitivity to gluten in the diet that affects multiple systems in the body in genetically predisposed individuals [1]. Currently, high-sensitivity serological tests make it possible to diagnose more cases of CD [2]. In a recent systematic review, the global seroprevalence of CD was approximately 1.4%, while the prevalence of biopsy-confirmed CD was 0.7% [3]. CD is known as one of the most common autoimmune disorders worldwide [1]. With the increasing awareness of medical caregivers, the overall prevalence of CD has been increasing in prospective regional studies [4]. The prevalence of CD may be influenced by age, gender, and geographic location [5]. Recent studies have found that the more socioeconomic problems, the deeper the CD symptoms [6]. A concerning epidemiologic issue regarding CD is that the recent studies have shown significant delay from clinical presentation to diagnosis [7]; therefore, considerable numbers of patients with CD remain undiagnosed due to its nonspecific clinical manifestations, hence the lower reported prevalence. In fact, in recent studies, most cases are asymptomatic and the clinical manifestations for symptomatic patients are heterogeneous [8].

The exact pathogenesis of CD remains unclear; however, the growing body of evidence has elucidated it to a large extent. With exposure to gluten, a group of alcohol-soluble proteins commonly found in cereals.

The clinical manifestations of CD are sorted into intestinal and extraintestinal, or classic and non-classic categories: the intestinal manifestations include weight loss, diarrhea, and malabsorption whereas extraintestinal category include a variety of manifestations such as osteoporosis, anemia, dermatitis herpetiformis, et cetera [9, 10].

A considerable amount of literature has focused on the connection between celiac disease and different obstetric complications including miscarriage, preeclampsia, and preterm birth. Previous studies managed to achieve invaluable findings; however, a large number of these studies lack standard definitions of celiac disease; some studies used outdated codes of International Classification of Diseases in the registrations of individuals with CD [11, 12]. Regarding the association between CD and miscarriage, while a number of studies reported no statistically significant odds of miscarriage for pregnancies with CD [13, 14], other studies including a recent systematic review and meta-analysis found a positive correlation between miscarriage and CD [8, 15]. Similarly, while a cohort study found no correlation between maternal CD and preterm delivery [16], other studies reported that women with CD had an increased risk of preterm labor [9, 17]. Nearly all of the studies found that celiac disease was not associated with significantly higher risk for preeclampsia [8, 17, 18].

Celiac disease is considered an autoimmune disease and is associated with a broad range of manifestations; therefore, the possibility of its complications including adverse pregnancy outcomes cannot be underestimated. Despite the higher occurrence of celiac disease in women and the potential risks associated with its delayed diagnosis that could impact their reproductive years, it is unsatisfactory that previous studies failed to provide standard definitions of celiac disease and did not reach an agreement regarding the relationship between celiac disease and pregnancy outcomes. Accordingly, we aimed to investigate the pregnancy outcomes in women with celiac disease.

## Methods

### Study design

In this retrospective cohort study conducted in Khorasan-e Razavi province, Iran, we evaluated the pregnancy outcomes in women with preexisting, biopsy-confirmed celiac disease (the exposed group) and those occurred in healthy women (the unexposed group) who had registered pregnancy records from 2017 to 2023.

### Participants, inclusion and exclusion criteria

**Fig. 1:**
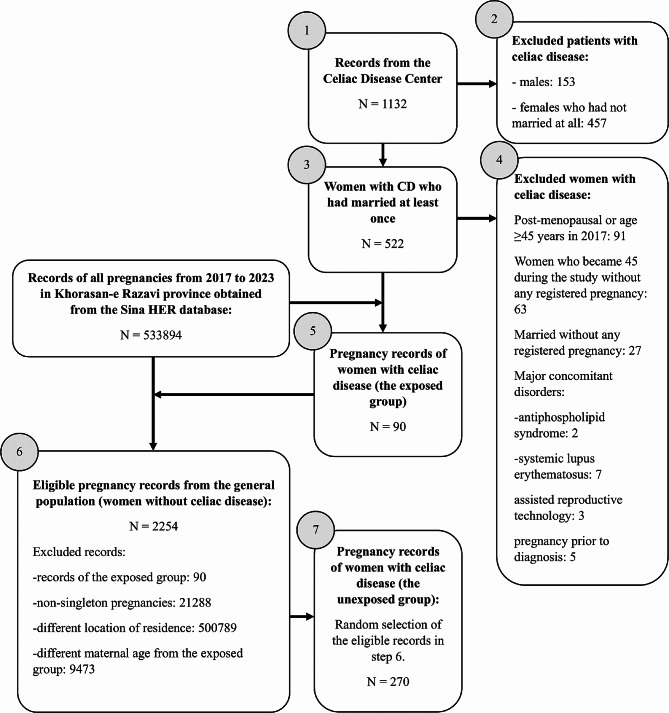
Data collection procedure flowchart

In this study, we focused on any terminated pregnancy from 2017 to 2023 that had been affected with maternal celiac disease (CD), irrespective of the time of diagnosis (be it before or after 2017). The terminated pregnancy applies to all pregnancies that were resulted in a final outcome including miscarriage, stillbirth, or delivery of a live baby. The outcomes and the follow up data were all available in the Electronic Health Registry (EHR) of Mashhad University of Medical Sciences called “Sina” [19]. The data collection procedure is shown in Fig. 1. It is emphasized that the pregnancy records, irrespective of exposure group, were only obtained from the Sina EHR database. This study design allowed complete data capture without requiring follow-up. The data on celiac disease status including the time of diagnosis and the histopathologic classification (Marsh-Oberhuber) classification was gathered from registrations in the Celiac Disease Center of Mashhad University of Medical Sciences which serves as the referral center for all patients with celiac disease across the Khorasan-e Razavi province. All registered patients were biopsy-proven. The matching was conducted to an exposed-unexposed ratio of 1:3, and the criteria for matching included [1] no prior history of maternal celiac disease or any other enteropathy, [2] maternal age at the time of conception (to a tolerance of two years compared to the maternal age of the exposed group), and [3] the location of residence (any record similar with any item of the exposed group). We were unable to objectively test the mothers of the unexposed group for celiac disease. Records with a maternal age ≥ 45 years at the first prenatal visit, high risk conditions (e.g. antiphospholipid syndrome, systemic lupus erythematosus, and thrombophilia), prior non-obstetric uterine surgery and any concurrent pregnancy at the time of data analysis were excluded. In addition, we excluded all pregnancies that were occurred through the assisted reproductive technology due to its higher risk for adverse pregnancy outcomes [20].

The main observed outcomes included normal delivery, low neonatal birthweight, postpartum hemorrhage, fetal anomalies, miscarriage, ectopic pregnancy, preeclampsia, preterm labor, gestational diabetes mellitus, gestational hypertension, gestational hypothyroidism, stillbirth and the mode of delivery (i.e. natural vaginal delivery or cesarean section). Based on the Celiac Disease Center principles, all patients with CD were offered free gluten-free material at the time of diagnosis confirmation, and every month thereafter. As a result, all patients with CD were supposed to stay on a gluten-free diet. The adherence of patients to the gluten-free diet was assessed using the patient-reported outcome measurement tool as well as serologic follow-up every six months. The mentioned inquiries were asked from the patients every three to six months and the data was available at the Celiac Disease Center database.

### Defining outcomes

Normal pregnancy outcome was defined as the completion of pregnancy course without any obstetric complications or mortality resulting in a normal delivery of a healthy infant after 37 weeks of gestation. Miscarriage was defined as unintended termination of pregnancy before 20 weeks of gestation. Ectopic pregnancy was defined as pregnancy occurred outside uterus with confirmed biomarker studies and ultrasonography. Preeclampsia was defined as blood pressure ≥ 140/90 accompanied by proteinuria (≥ 300 mcg in a 24-hour urine sample). Preterm labor was defined as spontaneous or medically indicated termination of pregnancy between 20 and 37 weeks of gestation. Stillbirth was defined as fetal demise after 20 weeks of gestation. Gestational diabetes mellitus was defined as impaired 1-hour oral glucose challenge test and a confirmatory 3-hour oral glucose tolerance test between 24 and 28 weeks of gestation. Finally, postpartum hemorrhage was defined as bleeding equal to or greater than 1000 mL, until 12 weeks following delivery, regardless of its route.

### Statistical analysis

We used Chi-square test in order to analyze the categorical variables between the exposed and unexposed groups. Continuous variables were analyzed using Student’s t-test or nonparametric Mann-Whitney U test. Kruskal Wallis H test analysis was used to evaluate the difference between the frequencies of adverse obstetric outcomes and the Marsh classification levels in women with CD. We used binary logistic regression analysis to calculate crude and adjusted odds ratios (aOR) with 95% confidence intervals (95% CI) in order to evaluate the associations of exposure to celiac disease and pregnancy outcomes such as miscarriage, stillbirth, preterm labor, and other items such as gestational diabetes mellitus and neonatal birthweight. We calculated the adjusted odds ratios (aORs) by taking into account maternal age [21]. This was necessary because adverse pregnancy outcomes tend to increase with maternal age, which could have affected our analysis.

## Results

Out of roughly 534,000 pregnancies included initially, 90 pregnancies in women with celiac disease were extracted (exposed group). After filtering all other entries based on the similarities in age (to a tolerance of two years), location of residence, and socioeconomic factors with the exposed group, 2254 records remained eligible for control (unexposed) group. In the process of filtering eligible records, we faced an unexpectedly high number of non-singleton pregnancies reaching over 21,000 records. A random sample of 270 pregnancies were selected as the unexposed group. Eventually, 360 singleton pregnancies including 90 pregnancies in the exposed group and 270 pregnancies in the unexposed group were studied. From 2017 to 2023, 30% of all women with confirmed CD who were registered in our clinic received medical care related to pregnancy. A total of 316 pregnancies, including 74 pregnancies in women with CD, were completed without any adverse obstetric outcome. The mean age of women with CD at diagnosis and the beginning of their pregnancies were 30.98±7.36 and 33.59±6.77, respectively (Table 1). None of the observed variables had significant differences between the exposed and unexposed groups. Sixty-two (68.9%) pregnancies occurred in the setting of a controlled celiac disease with a good response to a gluten-free diet. The logistic regression model was conducted to explore the effect of CD activity on pregnancy outcomes and resulted in no significant association in terms of all studied outcomes.

**Table 1:** General characteristics of included pregnancies

	Total (*N* = 360)	Pregnancies in women with celiac disease (*N* = 90)	Pregnancies in the control group (*N* = 270)	*p*-value
**Maternal age at** **the conception (mean [SD])**	31.04 (5.83)	33.59 (6.77)	32.68 (5.74)	0.65
**Delivery status (n [%])**				
Nullipara	179 (49.72)	39 (43.33)	140 (51.85)	0.10
Primipara	87 (24.16)	24 (26.66)	63 (23.33)	0.30
Multipara	94 (26.11)	27 (30.00)	67 (24.81)	0.18
**Prior miscarriage (n [%])**				
No miscarriage	273 (75.83)	68 (75.55)	205 (75.92)	0.52
Single miscarriage	64 (17.77)	15 (16.66)	49 (18.14)	0.44
Recurrent miscarriage	23 (6.38)	7 (7.77)	16 (5.92)	0.34
**Prior stillbirth (n [%])**				0.37
No	355 (98.61)	88 (97.78)	267 (98.89)	
Yes	5 (1.39)	2 (2.22)	3 (1.11)	
**BMI before pregnancy (mean [SD])**	24.73 (5.21)	23.30 (5.82)	25.30 (4.86)	0.01*
**Preexisting hypertension (n [%])**				0.06
No	358 (99.96)	88 (97.78)	270 (100)	
Yes	2 (0.5)	2 (2.22)	0 (0)	

BMI: body mass index

Six miscarriages occurred in the exposed group (6.66%) compared to eight (2.96%) in the unexposed group, which did not have a significant difference (aOR = 1.00, 95%CI = 0.91 – 1.10). Similarly, no significant difference was observed between the exposure groups regarding gestational diabetes mellitus (aOR = 1.07, 95%CI = 0.98 – 1.16). Moreover, no record of ectopic pregnancy was found in women CD (Table 2); interestingly, our analysis showed significantly lower odds of preeclampsia in women with CD when adjusted for maternal age at conception (aOR = 0.83, 95%CI = 0.69 – 0.99). The crude and adjusted odds ratios are shown in Table 3. The odds of preterm labor were not significantly higher in the exposed group (aOR = 2.49, 95%CI = 0.38-16.23). Six neonates (6.6%) in the exposed group had low birthweight (<2500 g) compared to 20 (7.4%%) in the unexposed group. We found no significant odds of postpartum hemorrhage, and no records of stillbirth was observed in the whole study. At last, the Kruskal Wallis H test analysis showed no significant difference between the frequencies of adverse obstetric outcomes and the Marsh classification levels in women with CD.

**Table 2:** Pregnancy outcomes in women with celiac disease and the control group

	Total (*N* = 360)	Pregnancies in women with celiac disease (*N* = 90)	Pregnancies in the control group (*N* = 270)	*p*-value
**Normal delivery (n [%])**	316 (87.77)	74 (82.22)	242 (89.62)	0.51
**Mode of delivery**				0.47
NVD	168 (48.55)	40 (47.62)	128 (48.85)	
CS	178 (51.44)	44 (52.38)	134 (51.15)	
**Neonatal birthweight**, **g (mean [SD])**	3184.72 (467.68)	3105.06 (435.16)	3211.80 (476.04)	0.34
Under 2500 g (n [%])	26 (7.2)	6 (6.66)	20 (7.4)	0.51
Above 2500 g (n [%])	334 (92.8)	84 (93.33)	250 (92.6)	
**Postpartum hemorrhage (n [%])**				0.01*
No	357 (99.16)	87 (96.7)	270 (100)	
Yes	3 (0.83)	3 (3.33)	0 (0)	
**Fetal anomalies (n [%])**				0.43
No	242 (99.2)	89 (99.99)	269 (99.99)	
Yes	2 (0.55)	1 (0.01)	1 (0.00)	
**Miscarriage (n [%])**				0.10
No	346 (96.12)	84 (93.33)	262 (97.03)	
Yes	14 (3.88)	6 (6.66)	8 (2.96)	
**Ectopic pregnancies (n [%])**				0.56
No	242 (99.45)	90 (100)	268 (99.26)	
Yes	2 (0.55)	0 (0)	2 (0.74)	
**Preeclampsia (n [%])**				0.33
No	355 (98.6)	90 (100)	270 (98.14)	
Yes	5 (1.3)	0 (0)	5 (1.8)	
**Preterm labor (n [%])**				0.51
No	345 (95.84)	86 (95.56)	259 (95.93)	
Yes	15 (4.16)	4 (4.44)	11 (4.07)	
**Gestational diabetes mellitus (n [%])**				0.13
No	342 (95)	83 (92.23)	259 (95.93)	
Yes	18 [5]	7 (7.77)	11 (4.07)	
**Gestational hypertension (n [%])**				0.39
No	351 (97.5)	87 (96.67)	264 (97/78)	
Yes	9 (2.5)	3 (3.33)	6 (2.22)	
**Hypothyroidism in pregnancy (n [%])**				
No	355 (98.61)	85 (94.45)	270 (100)	<0.001*
Yes	5 (1.38)	5 (5.55)	0 (0)	

^*^ The level of statistical significance was determined at 0.05

NVD: natural vaginal delivery; CS: cesarean section

**Table 3:** Crude and adjusted odds ratios for pregnancy outcomes

	Crude odds ratio (95% confidence interval)	Adjusted odds ratio (95% confidence interval)^a^	*p*-value^b^
Neonatal birthweight <2500 g	0.88 (0.34 – 2.29)	0.99 (0.92 – 1.06)	0.89
Postpartum hemorrhage	1	1.12 (0.91-1.38)	0.25
Fetal anomalies	2.56 (0.15 – 42.73)	0.89 (0.69 – 1.15)	0.37
Miscarriage	2.35 (0.79 – 7.04)	1.00 (0.91 – 1.10)	0.89
Ectopic pregnancies	1	0.94 (0.73 – 1.20)	0.64
Preeclampsia	1	0.83 (0.69-0.99)	0.04*
Preterm labor	1.15 (0.35 – 3.73)	1.00 (0.92 – 1.10)	0.86
Gestational diabetes mellitus	2.18 (0.81 – 5.89)	1.07 (0.98 – 1.16)	0.09
Gestational hypertension	1.51 (0.36 – 6.23)	0.99 (0.89 – 1.11)	0.98
Gestational hypothyroidism	1	0.95 (0.82 – 1.11)	0.58

^a^ adjusted for maternal age at the time of conception

^b^ calculated for the adjusted odds ratios

* The level of statistical significance was determined at 0.05

## Discussion

This retrospective cohort study analyzed pregnancy outcomes in women with biopsy-confirmed celiac disease (CD) compared to healthy controls in Northeast Iran between 2017 and 2023. To our knowledge, this is the first study to utilize the provincial health records in order to systematically investigate CD and pregnancy outcomes in an Iranian population. Our cohort covered over 530,000 pregnancies in the Khorasan-e Razavi province, which has a population exceeding 6 million people [22].

As we expected, the frequency of pregnancies was lower compared to the prevalence of CD in other studies. This low number of pregnancies might be a result of several factors including regional scope of our study rather than being nationwide, personal childbearing intentions of those with celiac disease due to maternal and fetal health concerns (especially the inheritance of CD), socioeconomic issues, or the celiac disease itself remaining undiagnosed during the study. Additionally, a considerable portion of the population reside in rural areas, where the level of medical care and the suspicion for CD are low; Yet, the reported prevalences of CD include people from all ages and all genders that are not necessarily contribute to the number of pregnancies.

The main outcomes to evaluate were obstetric events such as normal delivery, low neonatal birthweight, postpartum hemorrhage, fetal anomalies, miscarriage, ectopic pregnancy, preeclampsia, preterm labor, gestational diabetes mellitus, gestational hypertension, gestational hypothyroidism, stillbirth and mode of delivery.

Based on our results, 87.77% of all pregnancies, including 82.22% of those in women with CD, were completed without any adverse outcomes. The number of normal pregnancies was not significantly different between the exposed and unexposed groups (p-value = 0.09).

Notably, our study showed significantly lower odds of preeclampsia in women with celiac disease (aOR = 0.83, 95%CI = 0.69–0.99, p-value = 0.04). Our results were in contrast to the previous studies that found no significant association between CD and preeclampsia [16, 18]. In a comprehensive population-based study in England, Sultan et al. studied 363,930 pregnancies that resulted in live birth or stillbirth and found that women with CD had no higher risk for any pregnancy complications. On one hand, the methods used in our study was relatively similar; however, in our study, the number of pregnancies in women with celiac disease was disproportionately low due to several reasons. On the other hand, our result may reflect the results of case-control matching with multiple criteria, which was absent in the study by Sultan et al. [18]. Additionally, two recent meta-analyses investigated the association between CD and preeclampsia. The first study was done by Saccone et al. studying four studies with approximately 5,000 observed pregnancies including 258 pregnancies in women with CD [17], and found no significant association between CD and preeclampsia; however, they found that women with CD had significantly higher risk of composite pregnancy outcomes. The second was done by Arvanitakis et al. in which roughly 78,000 participants were studied, and resulted in no significant association between CD and preeclampsia [8].

Aside from preeclampsia, our results showed no significant association between CD and other pregnancy outcomes when adjusted for maternal age. These outcomes included low neonatal birthweight, postpartum hemorrhage, fetal anomalies, miscarriage, ectopic pregnancies, preterm labor, gestational diabetes mellitus, gestational hypertension and gestational hypothyroidism.

In a systematic review and meta-analysis, Arvanitakis et al. pooled the available data from observational studies and found that miscarriage had higher risk of occurrence in women with CD [8]. On one hand, our results would have been more accurate if we could include pregnancies prior to 2017. On the other hand, Arvanitakis et al. pooled data from studies that included assisted reproductive technology conceptions which could lead to higher miscarriage rates [23, 24]. Additionally, they included studies that used serologic markers to determine participants with celiac disease, that has been recently questioned by other studies for lacking a concrete diagnostic cut off value [2, 25]. Eventually, as mentioned by the authors, their work included highly heterogenous studies for assessment. Moreover, other recent case-control studies focusing on recurrent pregnancy losses and CD found no significant association [26]. As a results, further studies with well-defined exposure and outcomes are warranted for further investigation.

Our analysis showed no significant association between CD and ectopic pregnancy. This finding was in line with a recent systematic review by Talavera et al. that included two population-based studies [27–29]. These findings seem to be insufficient to draw a definitive conclusion, and may be confirmed with further studies.

In this study, no records of stillbirth were observed, suggesting that CD may not be associated with stillbirth. Concerning stillbirth, previous studies reported mixed results. For instance, Sultan et al. conducted a population-based study including 892 pregnancies in women with CD, and found no significant association. This finding was supported by Tata et al., who studied the primary care data of 1521 women with CD in a population-based cohort study. Similarly, Grode et al. found no significant association with diagnosed CD and stillbirth, however, when focused on pre-diagnosis CD cases, they found a significantly higher odds of stillbirth [29]. However, the meta-analysis by Arvanitakis et al. that included both cohort and case-control studies found significantly positive correlation between CD and stillbirth [8].

In addition, we found no records of postpartum hemorrhage in women with celiac disease. This finding was in line with previous literature in terms of postpartum hemorrhage, including population-based cohort studies [13, 16, 18, 28]. Surprisingly, our study did not find a significant association between celiac disease (CD) and preterm labor (p-value = 0.86). This finding was contrary to previous research. In a population-based retrospective cohort study including 212 pregnancies in women with CD, Abecassis et al. found a significant association between CD and preterm labor [9]. The same results were reported for undiagnosed CD by Ludvigsson et al., who conducted a population-based study from registered data, dating from 1964 to 2001; however, they were unable to find a significant association between diagnosed CD and preterm labor [12]. Both mentioned studies had used previous versions of International Classification of Diseases (ICD) codes to define celiac disease, and the validity of their diagnosis remains unclear. Additionally, the work by Moleski et al. found a significant association between CD and preterm labor [30]; however, this study was conducted as an online survey and the data was not originally collected from healthcare-associated centers. But, in line with our study, Sultan et al. found no significant association between CD and preterm labor [18]. Nevertheless, despite the possibly influential flaws in the methodology of the previous studies, the association of undiagnosed CD with adverse pregnancy outcomes including preterm labor cannot be excluded.

In addition to our main study goals, we evaluated the association between gestational diabetes mellitus and CD, and found no significantly higher number of GDM cases in women with celiac disease (aOR = 10.7, 95% CI = 0.98–1.16). This finding was also reflected by Elliot et al., who studied 2755 pregnancies in women with CD [16]. However, in an interesting study, Dalfrà et al. found that CD was not significantly associated with pregnancy outcomes among women with GDM [31].

Additionally, our results indicated that babies born to mothers with CD were not at higher odds of having low birthweights (i.e. under 2500 g). Some of the previous studies found significant association between neonatal low birthweight and CD. This finding was in contrast to previous studies. Khashan et al. found that the low neonatal birthweight was significantly higher in women with undiagnosed CD [11]. Similar results were reported for women with undiagnosed CD by Arvanitakis et al. and also by Sultan et al. [8, 18]. It seems that diagnosis of CD and the subsequent initiation of GFD may lower the incidence of neonatal low birthweight.

Eventually, it is worth noting that most of the literature focused on the influence of GFD and mucosal healing with the following resolution of malnutrition for explaining these significant differences between undiagnosed and diagnosed CD, which was challenged by Lebwohl et al. in a nation-wide study [32]. These findings may suggest other explanations rather than GFD for pregnancy complications in women with undiagnosed CD. Further research is recommended to clarify this matter.

## Conclusion

We found significantly lower odds of preeclampsia in women with CD. Our results showed no significant association between celiac disease and the rest of the observed adverse pregnancy outcomes including miscarriage, ectopic pregnancy, stillbirth, preterm labor, gestational diabetes mellitus, and having a baby with low birthweight. About 18% of pregnancies in women with CD were associated with a morbidity or fetal demise. Finally, the association between CD and miscarriage remained unclear.

## Strengths and limitations

Our work was initiated with the aim of conducting a systematic and corporate study on pregnancy outcomes in women with celiac disease, covering a relatively large population in Iran. To our knowledge, our study was the first study to utilize the medical records of the Department of Health and Provincial Health Center in such a large scale. Additionally, we only included women with confirmed biopsy results in order to avoid potential selection bias. Yet, our study would have been more accurate if we had been able to include more women with celiac disease. Our data may resemble the tip of an iceberg, as there are many people left undiagnosed with celiac disease due to its clinical complexity and the low medical attention from the general population. The definition of the unexposed group could have been more accurate if we were able to objectively test the selected mothers for celiac disease with serology and biopsy. Our work could have been more informative if we were able to include the time interval from the onset of celiac disease to pregnancy in our analyses; however, we faced an inevitable diagnostic delay due to the variable presentations as well as low clinical suspicion. Eventually, our study would be more inclusive if we had participants who were pregnant on and off the gluten-free diet.

## Data Availability

The data that support the findings of this study are available from Mashhad Department of Health and Provincial Health Center but restrictions apply to the availability of these data, which were used under license for the current study, and so are not publicly available. Data are however available from the authors upon reasonable request and with permission of Mashhad Department of Health and Provincial Health Center.
